# Sex differences in acute respiratory tract infections—multi-year analysis based on data from a large tertiary care medical center in Israel

**DOI:** 10.3389/fpubh.2025.1502036

**Published:** 2025-03-25

**Authors:** Victoria Peer, Michal Mandelboim, Menucha Jurkowicz, Manfred S. Green

**Affiliations:** ^1^School of Public Health, University of Haifa, Haifa, Israel; ^2^Department of Epidemiology and Preventive Medicine, Faculty of Medicine, School of Public Health, Tel Aviv University, Tel Aviv, Israel; ^3^Central Virology Laboratory, Chaim Sheba Medical Center, Ministry of Health, Ramat Gan, Israel

**Keywords:** sex differences, acute respiratory tract infections, adenovirus, influenza, rhinovirus, parainfluenza-3, human metapneumovirus, respiratory syncytial virus

## Abstract

**Introduction:**

Acute respiratory tract infections impose a considerable burden on the health services. The development of improved prevention and treatment measures requires a better understanding of the mechanisms of infection. Since sex has been shown to be an important biological variable in the immune response to infections, we aimed to assess sex differences in the incidence rates of respiratory infections.

**Materials and methods:**

We obtained data on cases hospitalized with diagnosed respiratory tract infections by sex and age group over a period of 11 years (2012–2022) from the Sheba Medical Center (SMC), the largest tertiary care medical center in Israel. Nasopharyngeal samples collected from the patients with symptoms of a respiratory tract infection were examined for adenovirus, influenza, rhinovirus, parainfluenza-3, human metapneumovirus (hMPV) and respiratory syncitial virus (RSV) in the Central Virology Laboratory and Viral RNA/DNA was extracted and tested using a real-time reverse transcription-PCR (rRT-PCR) assay. We calculated annual male to female incidence rate ratios (IRRs) which were combined over the period of the study using meta-analysis methodology.

**Results:**

There was a male excess in infection rates for all viruses, particularly in the youngest age groups of <0 and 1–4 years. Our analyses revealed that the influenza incidence rates were 42 and 28% higher in males in infants and toddlers. The male dominance was similar for adenovirus with 33 and 38% in infancy and age group 1–4. For RSV, the male to female IRR was higher at ages <1 and 1–4 (22 and 21% respectively). Males were more likely to be positive for rhinovirus in infancy and toddlers, by 40 and 25%, respectively.

**Conclusion:**

There is evidence of an excess incidence of respiratory diseases in males. The mechanism is unclear. Other than behavioral factors, there is a need to study the role of sex hormones and genetic factors.

## Introduction

Acute respiratory tract infections (ARTIs) are among the most common causes of morbidity worldwide (1, 2). These infections are caused by a variety of viruses (3, 4) and are responsible for a large proportion of physician visits (5, 6). The most common respiratory viruses that infect humans include adenoviruses, coronaviruses, human metapneumoviruses (hMPVs), rhinoviruses (RVs), influenza viruses, enteroviruses, parainfluenza virus (PIV), and respiratory syncytial virus (RSV) and COVID-19 (7).

There are varying clinical manifestations for each virus, occasionally with complications severe enough to require hospitalization (8, 9). Each of these viruses can be associated with wide range of symptoms, from influenza-like symptoms to acute respiratory distress syndrome that can lead to hospitalization, comorbidities and death (7). In the 20th century, influenza and pneumonia constituted the highest proportion of infectious disease deaths in the United States with a higher age-adjusted mortality rates in males than females (10). Rhinovirus is the factor that causes more than 50% of upper respiratory tract infections worldwide and together with RSV, RV is one of the leading causes of viral infections in infants (11). Influenza A viruses are responsible for seasonal epidemics and occasional outbreaks, and sporadic pandemics (12). The COVID-19 pandemic began with an outbreak of pandemic, caused the public health emergency of international concern with 7,079,129 confirmed deaths (13). Sex differences in selected infections including COVID-19 have been described (14).

There are reports indicating that males are overrepresented in incidence of infectious diseases (15–19) although this varies by disease and age. In a retrospective cohort Dutch study, the researchers found that female patients had a significantly higher incidence of respiratory symptoms compared with males (20). In another study on sex differences in the incidence of respiratory tract infections, males develop otitis media, croup and respiratory tract infections more frequently than females (21).

Many funding agencies in Europe and North America support and encourage researchers to consider sex as a biological variable in medical research (22). Sex is a biological variable that influences both the innate and adaptive immune systems. The presence of sex hormone receptors across multiple cell tissues and the pattern of expression of X-linked genes means that sex is a biological factor that influence the function of many physiological systems, including the immune system (23). The understanding of the ways in which sex impact on the immune response to respiratory virus infections is limited by the relative lack of data on this variable.

The objective of this study was to explore possible sex differences in the incidence of infections caused by selected respiratory viruses by age group, among patients hospitalized in a largest tertiary care hospital in Israel, between years 2012–2022.

## Methods

### Patients and samples

This study includes the results of laboratory tests of all nasopharyngeal samples collected from patients hospitalized at Sheba Medical Center due to respiratory illness and influenza-like symptoms between January 2012 and June 2022. Sheba is the largest tertiary care medical center encompassing six hospitals and centers, treating over 1.2 million people each year (24) which provides medical treatment and service to the population in the center of Israel. The source population (about 1.200.000 population) was estimated on the basis of the number of patients treated relative to the total number of patients receiving health care in Israel.

The samples were analyzed by the Central Virology Laboratory and tested for respiratory viruses including adenovirus, influenza, rhinovirus, parainfluenza-3, human metapneumovirus (hMPV) and respiratory syncytial virus (RSV).

If a patient had a culture sent simultaneously from the nose or pharynx or mouth or both the nose and throat and the same pathogen was detected in all tests, then the duplicates were removed and just one result was included. If a patient was diagnosed with infections due to different pathogens, each infection was recorded separately. If the patient was diagnosed with multiple infections during different time periods, all infections were included.

### Viral RNA extraction

All the samples were collected before treatment using Ʃ-Virocult Swab and Virus Transport Medium (MWE Medical Wire, England) and transported to the laboratory within 8 h from collection. Samples were stored at 4°C pending molecular analysis within 24 h of sample collection. Until January 2020, viral RNA/DNA was extracted using MagNA PURE 96 (Roch, Manheim, Germany). After January 2020, viral RNA/DNA extraction was performed using the STARMag Viral DNA/RNA 200C kit (Seegene Inc., South Korea).

### Real time PCR assay

Viral RNA/DNA of influenza, RSV, hMPV, parainfluenza, and adenoviruses were tested using real-time reverse transcription-PCR (rRT-PCR) assay (25, 26). The test master mix was prepared with Ambion Ag-Path master mix (Life technologies, United States) in multiplex reactions. Two different multiplex tests were performed: one reaction that included primers for influenza A (H3N2), influenza A (H1N1pdm), influenza B, RSV and the other containing primers for hMPV, parainfluenza and adenovirus. The RT-PCR assay was performed on an ABI 7500 instrument (Thermo Fisher Scientific, United Kingdom). From January 2020, rRT-PCR assays were also performed using the AllplexTM RV essential assay (Seegene Inc., South Korea) in a CFX Real-time PCR system (Bio-Rad, United States) (27, 28).

### Calculation of incidence rates and incidence rate ratios statistics

The annual male to female incidence rate ratios (IRRs) were calculated for each year and combined over the period of the study using meta-analytic methods. The grouping by age was as follows: infants <1, toddlers 1–4, middle childhood (5–9), puberty (10–14), reproductive age (15–44), middle adulthood (45–64) years, senior adulthood (65–79) and older adulths (80+). The annual disease incidence rate (IR) for each disease/age group was calculated by dividing the number of test positive patients by the size of the relevant total population for each year according to sex and age group expressed per 100,000 of population. The combined male to female incidence rate ratios over the years of the study were calculated using both the Mantel–Haenszel estimator for the fixed effects model and the Desmonian-Laird estimator for the random effects model. The results for both the fixed and random effects models are presented. The statistical significance level was set at 0.05 for both the fixed and random effects models (29). Analyses were carried out using WinPepi (Version 11.65, Aug, 2016).

### Ethics

All data collected from the Central Virology Laboratory were anonymous. The SMC institutional review board approved the research (Helsinki Number 0801-23-SMC).

## Results

The summary of isolates by age and sex for acute respiratory tract infections (for influenza, adenovirus, RSV, parainfluenza-3, rhinovirus and hMPV) between years 2012–2022 and midpoint population size is presented in Table 1. In absolute numbers, influenza was more common in older ages of 15–44, 45–64, 65–79, and 80+, while RSV was more common at ages of <1, 1–4, 15–44, 45–64, 65–80+. Adenovirus was more common at ages <1 and 1–4. Low incidence of rhinovirus, parainfluenza- 3 and hMPV cases was observed at ages 5–9 and 10–14 (Supplementary data on Table 3 shows data on the source population estimation for each calendar year by age and sex).

**Table 1:** Summary of the absolute numbers of isolates for influenza, adenovirus, RSV, rhinovirus, parainfluenza-3, and hMPV viruses at Sheba Medical Center in Israel, by age and sex, between the years 2012–2022.

Age groups	Males						Females					
	Influenza	Adenovirus	RSV	Rhinovirus	Parainfluenza-3	hMPV	Influenza	Adenovirus	RSV	Rhinovirus	Parainfluenza-3	hMPV
<1	132	516	692	264	189	116	88	366	534	178	148	68
1–4	369	883	601	362	248	208	274	607	471	273	207	159
5–9	146	99	90	92	42	62	98	84	52	67	31	41
10–14	69	44	42	47	47	28	41	22	32	39	13	25
15–44	545	128	190	185	139	99	727	98	166	140	120	102
45–64	629	84	259	158	210	127	531	72	233	118	144	128
65–79	915	94	320	231	211	200	703	59	305	164	184	189
80+	633	61	227	124	111	145	723	51	332	124	153	184

The data on the annual male to female annual incidence rates with 95% confidence intervals and *p*-values are presented in Table 2. At young ages between infancy and puberty (<1, 1–4, 5–9, 10–14) and at later ages (45–64, 65–79 and 80+), significantly higher incidence rates of influenza were observed in males than in females (IRR = 1.42, IRR = 1.28, IRR = 1.42, IRR = 1.60 and IRR = 1.25, IRR = 1.53 and IRR = 1.33 respectively). A similar trend was revealed for adenovirus. Male adenovirus incidence rates were higher in all ages with significant differences at ages <1, 1–4, 10–14, 65–79 and 80+ (IRR = 1.33, IRR = 1.38, IRR = 1.90, IRR = 1.88 and IRR = 1.82 respectively). Higher incidence rates of RSV in males were observed at all ages with the significant differences between sexes at ages <1 (IRR = 1.22),1–4 (IRR = 1.21), 5–9 (IRR = 1.65) and 65–79 (IRR = 1.24).

**Table 2:** Male to female incidence rate ratios (IRR) with 95% confidence intervals, for influenza, adenovirus, RSV, rhinovirus, parainfluenza-3, and hMPV viruses, by age groups, at the Sheba Medical Center, between the years 2012–2022.

	Age	Fixed effects model MH estimate of IRR	MH 95% CI	*p* value	Random effects model IRR	Random effects model 95% CI
Adenovirus	<1	1.33	1.16–1.52	0.00	1.34	1.11–1.63
	1–4	1.38	1.24–1.53	0.00	1.38	1.24–1.53
	5–9	1.12	0.84–1.50	0.44	1.20	0.89–1.62
	10–14	1.90	1.14–3.18	0.01	1.73	1.02–2.92
	15–44	1.29	0.99–1.67	0.06	1.28	0.98–1.67
	45–64	1.24	0.90–1.69	0.18	1.23	0.89–1.69
	65–79	1.88	1.36–2.60	0.00	1.76	1.20–2.59
	80+	1.82	1.26–2.64	0.00	1.77	1.20–2.60
hMPV	<1	1.61	1.20–2.17	0.00	1.56	1.13–2.15
	1–4	1.22	1.00–1.50	0.06	1.19	0.90–1.58
	5–9	1.44	0.97–2.13	0.07	1.48	0.86–2.56
	10–14	1.06	0.63–1.81	0.82	1.05	0.59–1.88
	15–44	0.96	0.73–1.26	0.75	0.96	0.73–1.28
	45–64	1.05	0.82–1.34	0.69	1.05	0.82–1.35
	65–79	1.25	1.02–1.52	0.03	1.25	1.02–1.52
	80+	1.20	0.97–1.49	0.10	1.19	0.89–1.58
Influenza	<1	1.42	1.08–1.86	0.01	1.38	1.02–1.86
	1–4	1.28	1.09–1.49	0.00	1.30	1.02–1.66
	5–9	1.42	1.10–1.83	0.01	1.40	1.08–1.81
	10–14	1.60	1.09–2.36	0.02	1.56	1.05–2.33
	15–44	0.74	0.66–0.83	0.00	0.74	0.65–0.84
	45–64	1.25	1.12–1.41	0.00	1.25	1.12–1.41
	65–79	1.53	1.39–1.69	0.00	1.54	1.32–1.80
	80+	1.33	1.20–1.48	0.00	1.33	1.20–1.48
Parainfluenza-3	<1	1.20	0.97–1.50	0.09	1.20	0.97–1.49
	1–4	1.14	0.94–1.37	0.18	1.14	0.93–1.41
	5–9	1.29	0.81–2.05	0.29	1.24	0.77–2.01
	10–14	3.35	1.84–6.10	0.00	2.75	1.51–5.04
	15–44	1.14	0.89–1.46	0.29	1.15	0.89–1.47
	45–64	1.54	1.25–1.91	0.00	1.52	1.20–1.93
	65–79	1.35	1.11–1.65	0.00	1.35	1.10–1.65
	80+	1.10	0.86–1.41	0.44	1.11	0.86–1.45
RSV	<1	1.22	1.09–1.37	0.00	1.22	1.09–1.37
	1–4	1.21	1.07–1.37	0.00	1.20	1.05–1.38
	5–9	1.65	1.17–2.31	0.00	1.59	0.91–2.77
	10–14	1.25	0.79–1.98	0.34	1.23	0.76–2.01
	15–44	1.13	0.92–1.39	0.26	1.12	0.91–1.38
	45–64	1.18	0.99–1.41	0.07	1.18	0.99–1.41
	65–79	1.24	1.06–1.45	0.01	1.23	1.05–1.44
	80+	1.04	0.88–1.23	0.65	1.02	0.79–1.31
Rhinovirus	<1	1.40	1.15–1.69	0.00	1.40	1.15–1.69
	1–4	1.25	1.07–1.47	0.01	1.28	0.98–1.67
	5–9	1.30	0.95–1.78	0.10	1.47	0.83–2.60
	14–10	1.15	0.75–1.76	0.52	1.15	0.75–1.76
	15–44	1.30	1.04–1.61	0.02	1.29	1.04–1.61
	45–64	1.40	1.11–1.78	0.01	1.40	1.11–1.78
	65–79	1.65	1.35–2.01	0.00	1.58	1.08–2.31
	80+	1.50	1.17–1.92	0.00	1.46	1.03–2.07

The incidence rates of parainfluenza-3 in males were higher in all age groups. Significant differences between males and females were demonstrated at ages 10–14, 45–64 and 65–79 (IRR = 3.35, IRR = 1.54 and 1.35 respectively). Higher male incidence rates in rhinovirus were observed at all ages with significant differences between sexes at ages <1, 1–4, 15–44, 45–64, 65–79, and 80+ (IRR = 1.40, IRR = 1.25, IRR = 1.30, IRR = 1.40, IRR = 1.65 and IRR = 1.50 for age groups respectively). The excess male hMPV incidence rates were significantly higher in infancy and senior adulthood (IRR = 1.61 and IRR = 1.25).

**Figure 1:**
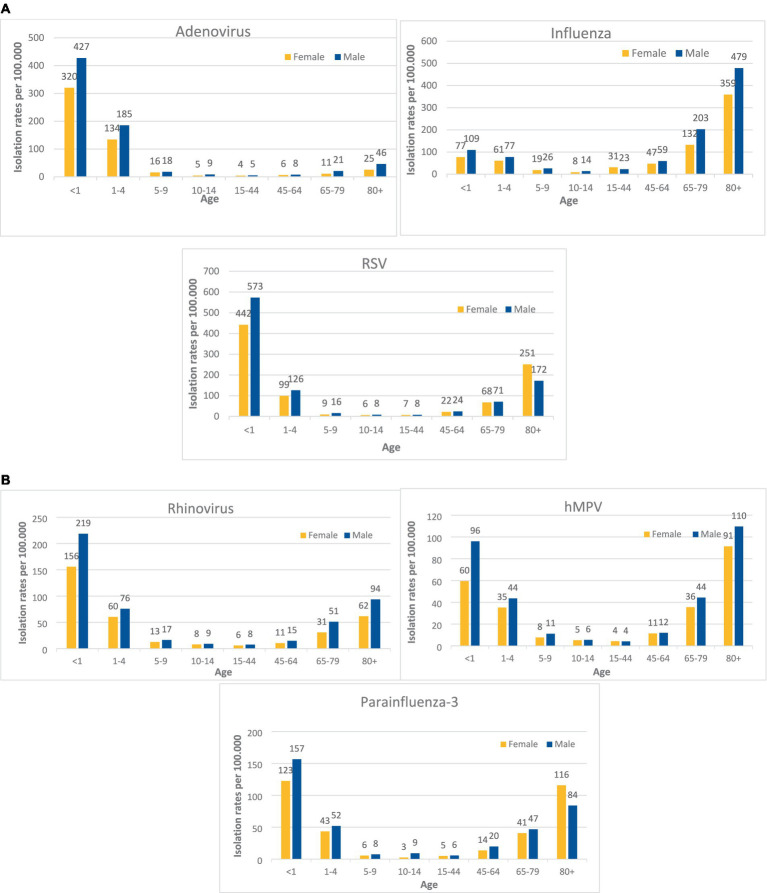
**(A)** Virus isolation rates per 100.000 population, at the Sheba Medical Center, by age and sex, 2012–2022. **(B)** Virus isolation rates per 100.000 population, at the Sheba Medical Center, by age and sex, 2012–2022.

Data on respiratory tract viruses Isolation rates per 100.000 population, by age and sex for years 2012–2022 are presented in Figures 1A,B. For all viruses, isolation rates per 100.000 population in young ages were higher in males than in females of the same age. For adenovirus and RSV isolation rates per 100.000 population at ages <1, 1–4 were as follows: 427 vs. 320, 185 vs. 134 (for adenovirus) and 573 vs. 442, 126 vs. 99 (for RSV) respectively by age groups. The same trend was observed for influenza, rhinovirus, parainfluenza-3 and hMPV. For young ages of <1 and 1–4 the isolation rates per 100.000 population at ages were as follows 109 vs. 77, 77 vs. 61 for influenza, 219 vs. 156,76 vs. 60 for rhinovirus, 157 vs. 123, 52 vs. 43 for parainfluenza-3 and 96 vs. 60, 44 vs. 35 for hMPV.

For all viruses, isolation rates per 100.000 population at ages 5–9, 10–14, 15–44, and 45–64 were low and the differences between sexes were negligible. Influenza virus isolation rate showed a male dominance for older age groups (except 15–44), 203 vs. 132 and 479 vs. 359 for ages 65–79 and 80+ respectively. Similar to the influenza virus, other pathogens isolation rates per 100.000 population at older ages (65–79, 80+) were higher in males in compare to females:44 vs. 36 and 110 vs. 91 for hMPV, 51 vs. 31 and 94 vs. 62 for rhinovirus, and 46 vs. 25 for adenovirus at age 80+. Parainfluenza 3 and RSV viruses isolation rates per 100.000 population were higher in females aged 80+ then in males of same age (116 vs. 84 and 251 vs. 172 respectively).

**Figure 2:**
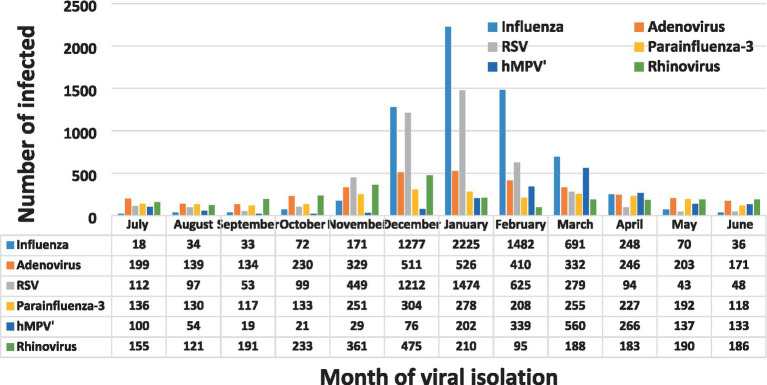
Number of isolated viruses at the Sheba Medical Center, by month, 2012–2022.

We observed seasonal variations in most of the viruses with the highest incidence during the winter months from November through March. hMPV and Adenovirus demonstrated a distinct seasonal variation with peaks in December to April and with decreased cases in September through November (Figure 2). Substantial levels of influenza virus and RSV activity and well-defined seasonality were demonstrated from December to March.

## Discussion

In this study of respiratory tract viruses isolated from patients from the largest hospital in Israel, we found that there was generally an excess of males in the incidence rates. Our analyses revealed that the influenza incidence rates were 42, 28, 42 and 60% higher in males in infants, toddlers, middle childhood and puberty, respectively. The trends of male dominance were similar for adenovirus with 33, 38, and 90% excess incidence in infancy, age group 1–4 and 10–14, respectively. For RSV male to female IRR was significantly higher at ages <1, 1–4, and 5–9 (22, 21 and 65% respectively). For parainfluenza-3 significant differences were evident in puberty and at ages 45–64, 65–79 with male preponderance of incidence of the disease (235, 54 and 35% respectively). Males were more likely to be positive for rhinovirus in infancy (40%), toddler age (25%) and in in older ages. Significant sex difference was demonstrated in hMPV incidence with 61% excess in males in infancy. In older ages (65+), the male: female IRR for almost all respiratory viruses was high and significant, except the RSV, parainfluenza-3 and hMPV at ages 80 + .

### Other studies

In a study that provides an overview of RSV prevalence in Iran from 1996 to 2013 the male–female ratio of RSV-positive patients was 1.5:1 (30). In the study on 16,018 patients with acute lower respiratory tract infection from a study in Buenos Aires, adenovirus infection was associated with age ≥ 12 months and male sex (31). A retrospective cohort study in Israel, on 1,227 children aged 0–23 months, showed that being a male was significantly associated with an increased incidence of RSV (male:female ratio of 1.35) (32).

There are contradictory data regarding the excess male incidence rates for acute respiratory tract infections. In Victoria, Australia, females were more likely to be positive for influenza and hMPV. There was no significant difference in sex distribution for the other viruses as RSV and parainfluenza (33). In study on hospitalized children<5 years old from Naples, Southern Italy, respiratory infection viruses demonstrated no substantial differences in sex and age (34).

In a large epidemiologic study in China, the overall viral detection rates were not significantly different between male and female patients, although female patients displayed higher rates for influenza virus, hMPV, and adenovirus (35). In a prospective cohort study among >1,000 children aged 0–35 months in Quebec City, Canada, with acute respiratory infection female sex was a risk factors for severe hMPV disease (36). In the study on children and adult patients n Córdoba, Argentina, of the 795 clinical specimens analyzed, the incidence of hMPV was higher in female patients in compared to male patients with ratio of 0.8 (37). In the prospective study of rhinovirus infections in adults (median age 50) recruited from 16 primary care networks in 11 European countries, the male to female ratio was 1:1.5 (38).

### Possible mechanisms

In this study we cannot provide mechanisms that may explain the sex differences in incidence of acute respiratory tract viral infections. Nevertheless, we can hypothesize that sex hormones and genetic differences are involved. We do not believe that in infancy and young children, behavioral differences between the sexes could affect the incidence rates of respiratory tract viral infections. We suggest that particularly in these ages, sex hormones and genetic factors could contribute a significant role in the sex differences observed. The mini-puberty period in infancy is associated with the transient sex-specific activation of the hypothalamic–pituitary-gonadal axis during the first 6 months of life in boys and during the first 2 years in girls. This phenomenon leads to a rise of sex hormones including estradiol, and testosterone and essential for physiological, including immune system body functions (39). Epigenetic modifications that affect X-linked gene expression could affect the immune response to infections in all ages (40).

At later ages, the differences between the sexes in incidence of respiratory tract infections may partly be explained by behavioral and social differences. Females may be more exposed to viruses when caring for children and in the workplace such as schools and kindergartens. Asymptomatic persons, especially children can be an important source of infection in their environment and could explain the increase of female disease incidence rate at childbearing ages of 15–44 and the older ages. In a study of Johnston SL et al. the viruses were isolated from asymptomatic subjects in 12% of children for rhinovirus (41). In Netherlands, the frequencies of subclinical infection and of asymptomatic subjects was high, 68%, for children at ages 0–15 (42). Occupations that involve considerable interaction with infected children can explain at least in part the differences in respiratory tract infection between ages15–44 and older.

In older ages, for males and females with the same chronological age, males prone to be biologically older (43). This also fits the established longer lifespan of females compared to males (44) which could be cause by biological factors, including genetic differences, sex hormones, age-related decline in testosterone (45, 46).

Some extrinsic factors such as preexisting immunity and microbiota could give at least partial explanation for sex differences in acute respiratory tract infections (47). The airway microbiome is involved in interactions between sex hormones, and immune systems, and it is highly influence on disease susceptibility. The number of shared microbiome species between the sexes was significantly lower than expected in the case in the respiratory tract (48).

Sex hormones affect the functioning of immune cells. Estrogen influence and enhance humoral responses to viral infection (49), Interferons (IFNs) and IRF5 express estrogen response elements (50, 51), are critical for protection from viral infection and cause the increased cytokine and chemokine production (52–56). Estradiol activity shows profound dose- and context-dependent effects on innate immune signaling pathways and promotes the production of interferons (57). Testosterone can decrease NK cell, neutrophil, and macrophage activity and reduce the production of pro-inflammatory cytokines, such as TNF-*α* (58). Testosterone diminishes pulmonary tissue inflammation, including virus-specific CD8+ T cells after the virus has been cleared (59). The pregnancy-related sex hormone concentrations and profound hormonal changes underlie many of the important immunological processes (60).

Genetic factors also could be implicated. There are many important genes, part of immune regulatory pathways, on the X chromosome, which escape the X inactivation event (61). Damaged genes on the X chromosome in males of significant immunological importance in males than in females, introducing sex-based bias and influencing the sex differences in immune response to infection (62, 63).

Existing clinical data show that the population, including children, young and aged individuals, males and females differ in vaccine-induced immune responses and protection. The study of Flanagan et al. (23) revealed that males are more likely to get vaccines, but following vaccination, females prone to develop higher antibody levels than do males. In Israel, male sex and older age were found to be associated with a higher willingness to be get vaccination (64).

### Seasonality

For all the viruses studied, the information about seasonality is important in vaccination and health services planning, especially when viruses co-circulate and putting pressure on hospitals. In Israel, influenza epidemics occur a little later than respiratory syncytial virus (December–April vs. November–March). Compared to other reports, in Israel, parainfluenza virus epidemics were found mostly in period between November to April (65). hMPV epidemics occurred in winter and spring. There is no demonstrated seasonality in adenovirus and rhinovirus epidemics, but there are peaks in the incidence of the disease in months November–March and November–December, respectively.

In Israel, during the COVID-19 pandemic a dramatic reduction in RSV and influenza virus activity was found. The circulation of both the parainfluenza and adenoviruses were mildly affected during the COVID-19 pandemic. For RSV, there was a seasonality shift with a delayed disease outbreak and greater weekly incidence rates (66, 67).

## Strengths and limitations of the study

We collected the molecular data from the analyses of respiratory samples over 11 years (2012 to 2022), which allowed us to obtain robust estimations of the incidence of these viruses in hospitalized patients in Israel. Our study population is a good representation of most of the population in Israel as Sheba Hospital, is the largest tertiary care medical center in Israel and in the entire Middle East (24) which provides medical treatment and service to the population in the center of Israel, treats over 1.200.000 patients annually.

The population seeks care in other primary or secondary hospitals and incidence estimates may be underestimated. There is no reason to believe that it will have a different effect on males and females and on sex ratios. In addition, this is a single-center study, thus, the generalizability of the results may be limited.

Underreporting is a known limitation, but it is unlikely to influence the findings since the objective was to study IRR and not absolute differences. The structure of the health system and society in Israel minimizes sex-related differences in access to medical service. We did not have information on possible exposure differences. In addition, the study was a single-center, thus, the generalizability of the results may be limited. In addition, cases with two or more pathogens were included more than once. This would affect the incidence rates, but the sex ratios should not be affected. It has been reported that the respiratory infection patterns and pathogen distributions changed significantly during the pandemic (68). Comparing age and sex differences in SARS-CoV-2 hospitalization and mortality with MERS-CoV, seasonal coronaviruses, influenza and other health outcomes revealed that age-specific sex differences in hospitalizations are largely similar across endemic and emerging infections (69). There is no reason to consider sex related differences in behavior or social distancing measures in children that would affect the infections transmission during COVID-19 pandemic (70).

## Conclusion

The results of this study add to the evidence of about a male dominance in the incidence rates of respiratory viruses, especially in young ages. Sex differences in the incidence of respiratory virus infections can contribute to a better understanding of the mechanisms of disease, and the development of improved prevention and treatment.

## Data Availability

The original contributions presented in the study are included in the article/Supplementary material, further inquiries can be directed to the corresponding author.
