# Supplementary material for: Sex differences in acute respiratory tract infections—multi-year analysis based on data from a large tertiary care medical center in Israel
Source: Front Public Health. 2025 Mar 25;13:1502036. doi: 10.3389/fpubh.2025.1502036 (PMC11975658; doi:10.3389/fpubh.2025.1502036)
Supplement: Supplementary file 1 [file Table_1.docx]

Supplementary data:

Table 3: The source population estimation for each calendar year by age and sex.

| **Male** | | | | | | | | | **Female** | | | | | | | |
| --- | --- | --- | --- | --- | --- | --- | --- | --- | --- | --- | --- | --- | --- | --- | --- | --- |
| **Year** | **0** | **1-4** | **5-9** | **10-14** | **15-44** | **45-64** | **65-79** | **80+** | **0** | **1-4** | **5-9** | **10-14** | **15-44** | **45-64** | **65-79** | **80+** |
| **2012** | 10320 | 39780 | 45240 | 41604 | 202068 | 88260 | 32256 | 10416 | 9852 | 37872 | 43020 | 39576 | 199560 | 94416 | 38784 | 16236 |
| **2013** | 10500 | 40740 | 46044 | 42504 | 205272 | 89220 | 34008 | 10668 | 9972 | 38808 | 43764 | 40476 | 202692 | 95244 | 40668 | 16560 |
| **2014** | 10704 | 41568 | 46980 | 43416 | 208452 | 90696 | 35700 | 10920 | 10104 | 39552 | 44688 | 41364 | 205764 | 96648 | 42456 | 16860 |
| **2015** | 10932 | 42276 | 48288 | 44172 | 211716 | 92304 | 37500 | 11196 | 10320 | 40152 | 45960 | 42072 | 208920 | 98172 | 44412 | 17232 |
| **2016** | 11076 | 43044 | 49608 | 44904 | 214944 | 93984 | 39360 | 11556 | 10464 | 40800 | 47232 | 42756 | 211956 | 99744 | 46464 | 17652 |
| **2017** | 11220 | 43800 | 50808 | 45756 | 218136 | 95736 | 41112 | 12012 | 10608 | 41424 | 48408 | 43524 | 214992 | 101376 | 48384 | 18252 |
| **2018** | 11292 | 44520 | 51960 | 46572 | 221580 | 97620 | 42828 | 12492 | 10716 | 42048 | 49488 | 44280 | 218244 | 103140 | 50220 | 18924 |
| **2019** | 11256 | 45120 | 53040 | 47544 | 225084 | 99816 | 44520 | 12912 | 10644 | 42672 | 50388 | 45228 | 221520 | 105192 | 52092 | 19452 |
| **2020** | 11040 | 45420 | 53964 | 48828 | 228288 | 102108 | 46224 | 13200 | 10428 | 42960 | 51180 | 46488 | 224436 | 107364 | 54048 | 19824 |
| **2021** | 11124 | 45312 | 54864 | 50136 | 231336 | 104472 | 47916 | 13392 | 10512 | 42876 | 51960 | 47748 | 227124 | 109656 | 56076 | 20076 |
| **2022** | 11280 | 45264 | 55884 | 51456 | 235428 | 107232 | 49788 | 13500 | 10620 | 42840 | 52848 | 49056 | 230736 | 112368 | 58380 | 20184 |
